# A PRESCRIPTION FOR WELLNESS: EXERCISE REFERRALS FOR PATIENTS AT A FEDERALLY QUALIFIED HEALTH CENTER

**DOI:** 10.1093/geroni/igz038.1913

**Published:** 2019-11-08

**Authors:** Gabriel Benavidez, Kelly Ylitalo

**Affiliations:** Baylor University, Waco, Texas, United States

## Abstract

Physical activity improves quality of life and prevents or delays chronic disease, but most adults in the United States are inactive. Consultation and planning with a health care provider, specifically with an exercise “prescription,” may increase physical activity, but utilization patterns and success of such programs are not well understood. This study assessed the initial 6 months of an exercise prescription program at a large, federally-qualified health center during 2018 whereby adult patients were referred via prescription to personalized health coaching by a fitness advisor. A census of all adults (n=512) who received an exercise prescription was combined with attendance data from the on-site exercise facility to classify patients as never attended, 1 to 3 visits, and ≥4 visits. Ordinal logistic regression was used to examine patient characteristics from the electronic health record that influenced exercise facility attendance. Only 30.2% of adults (mean age 44.7 years (SD 14.4)) completed ≥1 visit and 21.7% completed ≥4 visits. We identified no significant utilization differences by sex, race/ethnicity, body mass index, diabetes, hypertension, or coronary artery disease, but adults aged ≥60 years had almost twice the odds of ≥4 visits (OR=1.97; 95% CI: 1.18, 3.33; p=0.01) compared to younger patients. Many adult patients did not participate in the exercise prescription program, but older adults were more likely to participate. Exercise prescription programs with personalized health coaching may be useful for older adult patients receiving care at a federally-qualified health center. Future work will examine if or how exercise prescriptions impact chronic disease self-management.

